# Young adult concurrent use and simultaneous use of alcohol and marijuana: A cross-national examination among college students in seven countries

**DOI:** 10.1016/j.abrep.2021.100373

**Published:** 2021-09-04

**Authors:** Adrian J. Bravo, Mark A. Prince, Angelina Pilatti, Laura Mezquita, Matthew T. Keough, Lee Hogarth, Cross-Cultural Addictions Study Team

**Affiliations:** a Department of Psychological Sciences, William & Mary, USA; b Department of Psychology, Colorado State University, USA; c Universidad Nacional de Córdoba, Facultad de Psicología, Instituto de Investigaciones Psicológicas, IIPsi, CONICET, Córdoba, Argentina; d Department of Basic and Clinical Psychology and Psychobiology, Universitat Jaume I, Castelló de la Plana, Castellón, Spain; e Centre for Biomedical Research Network on Mental Health, Instituto de Salud Carlos III, Castelló de la Plana, Castellón, Spain; f Department of Psychology, York University, Canada; g School of Psychology, University of Exeter, United Kingdom

**Keywords:** College students, Substance use, Alcohol, Marijuana, Simultaneous use, Cross-cultural

## Abstract

**Introduction:**

Many young adults report frequent co-use of alcohol and marijuana, with some individuals engaging in simultaneous use (SAM; use of both substances within the same occasion resulting in an overlap of their effects) and others in concurrent use (CAM; use of both substances during a similar time period [e.g., past 30 days] but not within the same occasion). Emerging work demonstrates that SAM relative to CAM use places individuals at a greater risk for substance-related harms; however, these results primarily rely on U.S. samples. The goal of the present multi-country study was to examine prevalence rates of CAM and SAM use and examine differences in past 30-day SAM/CAM use on alcohol/marijuana substance-related outcomes among college students from seven countries.

**Methods:**

A total of 9171 (70.5% women; Mean age = 20.28, *SD* = 3.96) college students participated in the cross-sectional online survey study.

**Results:**

Among students who endorsed use of both alcohol and marijuana in the past 30-days (n = 2124), SAM use (75.8%) was far more prevalent than CAM use (24.2%). Moreover, ∼75% of students endorsed SAM use within each country subsample. Regression models showed that SAM vs. CAM use was associated with greater alcohol and marijuana use and negative consequences.

**Conclusions:**

College students from around the world endorse high rates of SAM use, and this pattern of co-use is associated with greater frequency of use and substance-related harms. On college campuses, SAM use should be a target of clinical prevention/intervention efforts and the mechanisms underpinning the unique harms of SAM need to be clarified.

## Highlights

• Young adults endorse high rates of simultaneous alcohol and marijuana (SAM) use.
• SAM use is associated with greater alcohol and marijuana use and related harms.
• Results were consistent among college students from seven different countries.

## 1 Introduction

Alcohol and marijuana use is highly prevalent worldwide, especially among young adults (Inter-American Drug Abuse Control Commission, 2019, Observatorio Español de las Drogas y las Adicciones, 2020, United Nations Office on Drugs and Crime, 2018). Among young adults, alcohol and marijuana use among college students is a significant public health concern because rates of use and the number of substance use-related negative consequences are high (Bravo et al., 2019, Bravo et al., 2019, Krieger et al., 2018). College attendance is associated with a high prevalence of alcohol use, particularly heavy drinking (Krieger et al., 2018, Linden-Carmichael and Lanza, 2018, Patrick and Terry-McElrath, 2017), and with the onset and escalation of marijuana use (Miech et al., 2017, Suerken et al., 2014).

### 1.1 Simultaneous and concurrent use of alcohol and marijuana

Simultaneous alcohol and marijuana (SAM) use is defined as using both substances within the same occasion, resulting in an overlap of their effects (Sokolovsky et al., 2020, Subbaraman and Kerr, 2015). SAM use is prevalent among young adults (Linden-Carmichael et al., 2019, Terry-McElrath and Patrick, 2018), including college students (Cadigan et al., 2019, Looby et al., 2021, White et al., 2019). For example, one study among college students (*N* = 1389) who reported use of alcohol and marijuana in the past year found high rates of past year (73%), past 3-month (58.9%), and past month (49.8%) SAM use (White et al., 2019). In addition to being prevalent, SAM use is associated with an increased risk of substance use and experiencing negative consequences related to use. Individuals who endorse SAM use exhibit greater frequency and quantity of alcohol use compared to those who only drink alcohol (Linden-Carmichael et al., 2019, Subbaraman and Kerr, 2015). Moreover, individuals that engage in SAM use are significantly more likely to experience negative consequences (Cummings et al., 2019, Linden-Carmichael et al., 2020, Jackson et al., 2020), including driving under the influence (Terry-McElrath et al., 2014, Subbaraman and Kerr, 2015).

An underlying question is whether these differences emerge because these two substances are used simultaneously or due to the concurrent consumption, even when they are not used in the same occasion/session. Research on SAM (the use of both substances at the same time such that effects overlap) and concurrent alcohol and marijuana (CAM; the use of both substances during a similar time period [e.g., past 30 days] but not within the same occasion) use suggest that although SAM use seems to have a more deleterious effect, both patterns of use contribute to increased vulnerability for young adults (compared to single substance using groups) to experience negative consequences. Among a sample of college students that reported use of alcohol and marijuana in the past year, Jackson et al. (2020) found that students that engaged in SAM use experienced more negative consequences than students that engaged in CAM use; yet, when examining each negative consequence and controlling for relevant variables (e.g., consumption rates), only blackouts were significantly different between students that endorsed SAM vs. CAM use. In another study among first year college students with prior alcohol use, Cummings et al. (2019) found that students who endorsed either SAM or CAM use engaged in greater alcohol use and experienced more alcohol-related negative consequences, including risky sexual behavior, than students who only drank alcohol. Students that endorsed SAM use also exhibited greater frequency of blackouts and academic problems than students that endorsed CAM use (Cummings et al., 2019).

In examining marijuana outcomes, Looby et al. (2021) found that among college students that endorsed using both alcohol and marijuana at least once in the past month, students who endorsed SAM use reported more marijuana use and negative consequences than students reporting CAM use. Moreover, Sokolovsky et al. (2020) obtained daily reports on alcohol and marijuana use among college students that endorsed past year marijuana and alcohol use and found that on days featuring use of both substances, compared to days were alcohol or marijuana were used exclusively, young adult college students experienced more negative consequences.

### 1.2 Cross-national examination of SAM and CAM use

Most of the research on SAM or CAM use has been focused in North-America, particularly using U.S., samples. This is unfortunate, considering marijuana and alcohol use are pervasive behaviors worldwide (CICAD, OAS, 2019; Observatorio Español de las Drogas y las Adicciones, 2020; UNDOC, 2018). Cross-cultural research of these behaviors and associated harms is a remaining challenge, and may be especially relevant among countries with different consumption policies, as they could help to identify protective measures that could be implemented in the most vulnerable countries. For example, the legal age to access alcohol usually differs across countries (e.g., 18 in Spain, Uruguay, Argentina, South-Africa and England; 19 in most provinces and territories in Canada; and 21 in the U.S). Marijuana policies differ even more between countries. In the U.S., laws determining legal status of marijuana, availability of marijuana, and acceptability of use vary from state to state, leading to differences in use and negative consequences (Hasin et al., 2017). Further recreational marijuana use is legal in Uruguay and Canada and illegal (with differing regulations for medicinal purposes) in Spain, Argentina, England, and South Africa (see Table 1 for a description of various alcohol/marijuana polices among countries assessed in the present study).

**Table 1:** Alcohol and Marijuana Policies across Data Collection Cites during Data Collection Period (2019–2020).

Country	Legal Access to Alcohol	Legal Access to Recreational Marijuana	Medical use of Marijuana	Other Relevant Cultural Information about Drug Policies
Argentina	18 years old	Decriminalized for private use but personal cultivation prohibited	Regulated	
Canada	19 years old, with the exception of Quebec, Manitoba and Alberta (where it is 18)	19 years old in most of the country, with the exceptions being Alberta (age 18) and Quebec (age 21).	Regulated	
England	18 years old	Illegal	Regulated	
South Africa	18 years old	Legal for possession and cultivation, but not for sale	Legal but not regulated	
Spain	18 years old at state level (and in the autonomous community included in the present research)	Buying, selling, and use are illegal in public settings. Decriminalized for private growing and use.	Not regulated	There are ‘cannabis social clubs’ (CSC) where the “private” sale and consumption is allowed at 18 or 21
U.S.	21 years old	Colorado (recreational cannabis is legal for those aged 21 + and can be purchased at registered dispensaries throughout the state). Recreational marijuana use is illegal in New York, New Mexico, and Virginia at time of data collection.	Regulated in the majority of the states (and all the states included in the present research)	Marijuana Laws vary across states.
Uruguay	18 years old	Buying, cultivating (up to six plants) and recreational use are legal.	Legal but not totally regulated	

*Note*. Access is defined as legal age to purchase alcohol or marijuana.

Moreover, college environment (e.g., living on campus, academic schedules and social organizations like fraternities and sororities), which represents a risk factor for substance use (Merrill & Carey, 2016), dramatically differs across countries. Living on campus and social organizations, distinct features of colleges in North-America, are largely absent in other countries. As substance use behaviors are sensitive to social, cultural, and regulating factors (Sudhinaraset et al., 2016), all these cross-cultural differences might impact in the prevalence and level of negative consequences associated with SAM and CAM use.

### 1.3 Purpose of the present study

The purpose of the present study was to examine prevalence rates of CAM and SAM use across college students from seven countries. Further, we examined CAM vs SAM use status as a predictor on variables of alcohol use, alcohol-related problems, marijuana use, and marijuana-related problems and examined if these effects were similar across countries. In line with prior research, we expected that students who report SAM use compared to CAM use to report higher marijuana and alcohol use and more negative consequences. Comparing results across countries were largely exploratory and aimed to test the universality of our findings. Confirming the predicted results would emphasize simultaneous polysubstance use as an important risk process underpinning substance related problems.

## 2 Method

### 2.1 Participants and procedures

Participants were college students recruited to participate in an online survey from the U.S. (five universities across four states: Colorado, New Mexico, New York, Virginia), Argentina (two public universities in the Cordoba region), Spain (one university located in the autonomous community of Valencia), Uruguay (one university located in the largest city of the country, situated on the southern coast of Uruguay), England (one university located in the city of Exeter), Canada (two universities; located in the provinces of Ontario and Manitoba) and South Africa (one university located in Cape Town) between February 2019 and March 2020. A total of 9171 (70.5% women; Mean age = 20.28, *SD* = 3.96) college students participated in the study (see Table 2 for demographics across countries).

**Table 2:** General demographics.

	Total	USA	Canada	South Africa	Spain	Argentina	Uruguay	England
Total Sample Size	*n* = 9171	*n* = 4265	*n* = 1655	*n* = 811	*n* = 764	*n* = 1037	*n* = 184	*n* = 455
Age (Mean, SD)	20.28 (3.96)	19.62 (3.27)	19.91 (4.09)	20.34 (2.21)	21.01 (3.06)	22.37 (5.23)	26.69 (7.48)	19.15 (3.42)
Gender								
Men	28.7%	32.5%	32.3%	16.2%	29.7%	23.2%	12.0%	18.7%
Women	70.5%	66.8%	66.5%	81.9%	70.2%	76.5%	88.0%	79.8%
Other/Missing	0.8%	0.7%	1.3%	2.0%	0.1%	0.3%	0.0%	1.5%
Education*								
First Year (Freshman)	–	54.9%	66.0%	37.2%	25.7%	32.2%	12.5%	97.8%
Second Year (Sophomore)	–	23.7%	23.0%	27.3%	34.0%	25.7%	22.3%	1.3%
Third Year (Junior)	–	13.1%	6.7%	25.3%	17.1%	17.6%	25.0%	0.7%
Four Year (Senior)	–	7.8%	2.2%	7.6%	14.4%	10.4%	20.1%	–
Fifth/Sixth/Seventh Year	–	–	1.3%	1.2%	2.6%	7.2%	1.6%	–
Other or Missing		0.5%	0.7%	1.3%	6.2%	6.9%	19.5%	0.2
	Total	USA	Canada	South Africa	Spain	Argentina	Uruguay	England
Alcohol and Marijuana Use – Lifetime	*n =* 9171	*n =* 4265	*n* = 1655	*n =* 811	*n =* 764	*n =* 1037	*n =* 184	*n =* 455
Never consumed either	11.3%	14.7% _a_	11.1% _b_	12.9% _a,b_	9.6% _b_	3.2% _c_	2.2% _c_	2.2% _c_
Only consumed alcohol	36.2%	32.5% _a_	43.1% _b_	26.5% _c_	38.6% _b,d_	36.3% _a,d_	35.9% _a,b,c,d_	59.1% _e_
Only consumed marijuana	1.1%	1.1% _a_	0.7% _a_	4.1% _b_	0.1% _a_	0.2% _a_	0.0% _a_	0.2% _a_
Has consumed both at least once	51.4%	51.6% _a_	45.1% _b, c_	56.5% _a, d_	51.7% _a, c_	60.4% _d_	62.0% _a, d_	38.5% _b_
	Total	USA	Canada	South Africa	Spain	Argentina	Uruguay	England
Alcohol and Marijuana Use – 30 day	*n =* 4715	*n =* 2201	*n* = 746	*n* = 458	*n* = 396	*n* = 626	*n* = 114	*n* = 175
No use in past 30 days	10.3%	10.5% _a_	10.5% _a_	9.6% _a_	10.9% _a_	11.2% _a_	14.0% _a_	2.3% _b_
Only used alcohol in past 30 days	37.8%	31.6% _a_	38.6% _b_	41.5% _b_	59.7% _c_	34.8% _a,b_	39.5% _a,b_	63.4% _c_
Only used marijuana in past 30 days	4.5%	6.0% _a_	4.3% _a,b_	2.4% _b_	2.3% _a,b_	4.0% _a,b_	3.5% _a,b_	1.1% _a,b_
Used both at least once in past 30 days	47.3%	52.0% _a_	46.6% _a_	46.5% _a_	27.1% _b_	50.0% _a_	43.0% _a, c_	33.1% _b,c_
	Total	USA	Canada	South Africa	Spain	Argentina	Uruguay	England
Concurrent vs Simultaneous Use – 30 day	*n =* 2124	*n =* 1129	*n =* 335	*n =* 193	*n =* 101	*n =* 266	*n =* 42	*n =* 58
Only concurrent Use	24.2%	26.0% _a_	26.6% _a,b_	23.3% _a,b_	23.8% _a,b_	16.9% _b_	11.9% _a,b_	20.7% _a,b_
Used both simultaneously	75.8%	74.0% _a_	73.4% _a,b_	76.7% _a,b_	76.2% _a,b_	83.1% _b_	88.1% _a,b_	79.3% _a,b_

*Note*. *Education was assessed differently for each country. USA = United States of America. Significant differences in prevalence rates across countries were determined by differences in proportions using a Z-test with a Bonferroni correction. Each subscript letter denotes a subset of country categories whose column proportions do not differ significantly from each other (i.e., if countries share the same subscript then there was no statistically significant difference detected).

Across all sites, students completed the same core battery of measures translated into the native language. To minimize burden on participants, we utilized a planned missing data design (i.e., matrix sampling, Graham et al., 2006, Schafer, 1997) which has been used in other large multi-site college student studies (e.g., Bravo et al., 2018). Specifically, each participant received and completed a battery of core measures that focused on substance use (i.e., alcohol, marijuana, opioids, stimulants and other drugs), addictive behaviors (gambling, internet use, gaming behavior), and a measure of mental health. After completing the core measures, each participant received a random sample of 12 measures from a larger pool (17 total measures) that assessed rumination, personality (i.e., impulsivity-like traits, Big Five personality traits), antisocial behavior, mindfulness, distress tolerance, self-regulation, emotion regulation, food addiction, subjective happiness, childhood trauma and experiences, and driving under the influence.

For the U.S. sites, Canadian sites, England site, and South African site students were recruited from Psychology Department pools and received research participation credit. In Argentina and Uruguay students were recruited disseminating an invitation through online social networks, e-mail listings and flyers (only in Argentina). In Uruguay and Argentina, participants who completed the survey took part in a raffle of prizes (Uruguay: 10 cash prizes [each of ≈US$ 20 at the time]; Argentina: 25 prizes each one of ≈US$ 10 at the time [10 vouchers for a bookstore and 15 cash prizes]). In Spain an email was sent to all the students of the university inviting them to participate in the research. The participants received 5 euros for completing the survey, which was available until the funds were consumed. Study procedures were approved by the institutional review boards (or their international equivalent) at the participating universities.

### 2.2 Measures

#### 2.2.1 Alcohol use indicators

Alcohol use was assessed using several indicators: an indicator of past 30-day alcohol use frequency, past 30-day frequency of getting drunk, past 30-day frequency of getting sick from drinking, past 30-day binge drinking frequency (i.e., past 30-day frequency of drinking 4+/5+ standard drinks in a period of two hours or less for women/men), an indicator of typical frequency of alcohol use, and an indicator of typical quantity of alcohol use. Participants were first presented with a visual guide about typical drinks (specific to each country), in order to help orient them to Standard Drink Units (SDUs). We assessed typical alcohol frequency and quantity using a grid such that each day of the week was broken down into six 4-hour blocks of time (12a-4a, 4a-8a, 8a-12p, etc.) and participants were asked to report at which times they consumed alcohol during a “typical week” in the past 30 days, as well as the number of standard drinks consumed during that time block. The measure was translated into Spanish for students in Argentina, Spain, and Uruguay. We calculated typical frequency of alcohol use by summing the total number of time blocks for which they reported using alcohol during the typical week (ranges: 0–42). We calculated typical quantity of alcohol use by summing the total number of SDUs consumed across time blocks during the typical week. To make accurate comparisons across countries, the total number of SDUs consumed (summed) were transformed into grams of alcohol taking into account country specific SDU rates based on grams of alcohol (quantity estimates > 3SDs above the mean were Winsorized).

#### 2.2.2 Marijuana use indicators

Marijuana use was assessed using several indicators: an indicator of past 30-day marijuana use frequency, an indicator of typical frequency of marijuana use, and an indicator of typical quantity of marijuana use. Participants were presented with a visual guide showing different amounts of marijuana in grams. Typical marijuana use frequency and quantity was assessed using the Marijuana Use Grid (MUG; Pearson & Marijuana Outcomes Study Team, 2021). The measure was translated into Spanish for students in Argentina, Spain, and Uruguay. Specifically, each day of the week was broken down into six 4-hour blocks of time (12a-4a, 4a-8a, 8a-12p, etc.), and participants were asked to report at which times they used marijuana during a “typical week” in the past 30 days as well as the quantity of grams consumed during that time block. We calculated typical frequency of marijuana use by summing the total number of time blocks for which they reported using during the typical week (ranges: 0–42). We calculated typical quantity of marijuana use by summing the total number of grams consumed across time blocks during the typical week (quantity estimates > 3SDs above the mean were Winsorized).

#### 2.2.3 Alcohol-related and Marijuana-related problems

Past 30-day alcohol-related problems were assessed using the 24-item Brief-Young Adult Alcohol Consequences Questionnaire (B-YAACQ; Kahler et al., 2005) and its Spanish version for students in Argentina, Spain, and Uruguay (Pilatti et al., 2014). Past 30-day marijuana-related problems were assessed using the 21-item Brief Marijuana Consequences Questionnaire (B-MACQ; Simons et al., 2012) and its Spanish version for students in Argentina, Spain, and Uruguay (Bravo et al., 2019). For both measures, we summed all items to create a composite score reflective of the number of distinct alcohol/marijuana problems experienced in the past 30-days.

#### 2.2.4 CAM vs SAM use

Students who reported consuming both alcohol and marijuana at least once in the past 30-days were asked to report how often (i.e., how many days) their alcohol and marijuana use was simultaneous. Specifically, these students were instructed to “indicate in the last 30 days how often you used alcohol and marijuana simultaneously (i.e., during the same use session)”. Students were instructed to enter zero days if they did not use these substances simultaneously. In order to make comparisons between students endorsing CAM vs. SAM use, students that reported at least one day of simultaneous use were coded as the SAM use group and students that reported never engaging in simultaneous use in the past 30-days (i.e., reported zero days) were coded as the CAM use group.

### 2.3 Statistical analyses

To test study aims, we first examined differences in prevalence rates of distinct alcohol/marijuana use patterns across countries. Significant differences in prevalence rates across countries were determined by differences in proportions using a Z-test with a Bonferroni correction. Next, we compared alcohol and marijuana use and consequences among students reporting past 30-day CAM vs. SAM use using a series of regression models. Most outcomes were best treated as highly skewed and over dispersed count variables. These outcomes were modeled using negative binomial regression (Hilbe, 2011). The two exceptions were alcohol and marijuana quantity consumption variables, which were best modeled as log-transformed in ordinary least squares regression models. For both negative binomial and log transformed outcomes, unstandardized regression coefficients (i.e., estimates) can be exponentiated to ease interpretation. In negative binomial models, exponentiation results in a Rate Ratio (RR), which is interpreted as the predicted percent change in the count for a 1-unit change in the predictor. Similarly, an exponentiated unstandardized regression coefficient from a log-transformed outcome can carry a similar interpretation with one additional step. That is, if you subtract 1 from the exponentiated value, the result is a decimal that is interpreted as the percent change in the outcome for a 1-unit change in the predictor. Typically, the RRs and the exponentiated log-transformed unstandardized regression coefficients minus 1 are multiplied by 100, for interpretation yielding a percent.

In all models described above we controlled for age and gender and used a binary predictor comparing CAM to SAM use. For models predicting marijuana/alcohol problems, marijuana/alcohol frequency of use was entered as a covariate. We ran two sets of models. The first set of models included the entire analytic sample (see below). The second set of models utilized a mixture modeling framework with a known class specification to run a type of multi-group analysis across countries to allow for the estimation of separate effects for each country within the same model. All models were run in Mplus version 8 (Muthen & Muthen, 2019). For analyses that include at least one count outcome, the default estimator is maximum likelihood with robust standard errors and missing data is handled using full information maximum likelihood, both are best practices for handling non-normal count data with missing data (cf. Yuan & Zhang, 2012). Finally, due to the large number of statistical tests and our relatively large sample size we chose to use 99% confidence intervals (CIs) to indicate significance. Note that for RRs CIs should not include 1 to be considered statistically significant and for the 1- exponentiated unstandardized regression coefficients the CIs should not include 0 to be considered statistically significant.

## 3 Results

### 3.1 Prevalence rates

Prevalence rates of distinct marijuana and alcohol use patterns in the total sample and across countries is presented in Table 2. Within the total sample (*n* = 9171), 11.3% (*n* = 1038) of students reported never consuming alcohol nor marijuana at least once in their lifetime, 36.2% (*n* = 3321) of students reported only consuming alcohol at least once in their lifetime, 1.1% (*n* = 97) reported only consuming marijuana at least once in their lifetime, and 51.4% (*n* = 4715) reported consuming alcohol and marijuana (i.e., have used both substances) at least once in their lifetime. Among individuals who have tried both substances (*n* = 4715), 10.3% (*n* = 486) reported no alcohol or marijuana use in the past 30 days, 37.8% (*n* = 1783) reported consuming only alcohol in the past 30-days, 4.5% (*n* = 214) reported consuming only marijuana in the past 30-days, and 47.3% (*n* = 2232) reported consuming both alcohol and marijuana at least once in the past 30-days.

Of the 2232 students that reported consuming both alcohol and marijuana at least once in the past 30-days, 2124 reported (95.16% response rate) how often (i.e., how many days) their alcohol and marijuana use was simultaneous. Among the 2124 students, 24.2% (*n* = 514) reported never using alcohol and marijuana simultaneously (coded as the CAM use group in analyses), compared to 75.8% (*n* = 1610) who reported simultaneously using alcohol and marijuana during the same use session at least once in the past 30 days (coded as SAM use group in analyses). In examining differences across countries, substance use patterns were largely consistent. For example, roughly 75% of students reported SAM use at least once in the past 30 day within each country subsample (exception being Argentinean and Uruguayan students, whom reported a higher percentage).

### 3.2 Model results

Results for comparisons of those who reported SAM use to those who reported CAM use for the entire sample, are presented in Table 3. Results for the models separated by country are presented as Supplemental Tables 1–7. Among alcohol outcomes, those who reported SAM use reported between 34% and 42% more alcohol use compared to those who reported CAM use, and all but one of the effects were statistically significant (i.e., number of times being sick in the past 30 days). Similarly, those who reported SAM use reported 25% more alcohol-related problems on the BYAACQ compared to those who reported CAM use. Regarding marijuana use and consequences outcomes, those who reported SAM use also reported more marijuana use (94% more past 30-day use frequency, 98% greater quantity, 110% higher frequency) and 52% more marijuana-related problems on the B-MACQ, compared to those who reported CAM use.

**Table 3:** Negative binomial regression models among those reporting past 30-day alcohol & marijuana concurrent use vs. simultaneous use in total sample*.*

	Concurrent Use (*n* = 514)	Simultaneous Use (*n* = 1610)	Negative Binomial Regression Models Results (0 = concurrent; 1 = simultaneous)			
***Alcohol Use Indicators***	*M* (*SD*)	*M* (*SD*)	*Estimate*	*RR*	*0.5% CI*	*99.5% CI*
Use Frequency Last 30 Days	5.16 (4.71)	7.04 (5.36)	0.29	**1.34**	**1.20**	**1.51**
Drunk Frequency Last 30 Days	2.61 (3.29)	3.61 (3.65)	0.35	**1.41**	**1.22**	**1.65**
Sick from Drinking Frequency Last 30 Days	0.49 (1.98)	0.62 (1.35)	0.29	1.34	0.85	2.10
Binge Frequency Last 30 Days	2.24 (3.26)	3.16 (3.71)	0.34	**1.40**	**1.18**	**1.68**
Typical Quantity*	121.20 (106.11)	166.82 (134.43)	0.35	**0.42**	**0.26**	**0.60**
Typical Frequency	3.19 (2.68)	4.35 (3.33)	0.29	**1.34**	**1.19**	**1.5**
***Alcohol-related Consequences***	*M* (*SD*)	*M* (*SD*)	*Estimate*	*RR*	*0.5% CI*	*99.5% CI*
B-YAACQ – Total Score	4.61 (4.11)	6.26 (4.67)	0.23	**1.25**	**1.12**	**1.41**
***Marijuana Use Indicators***	*M* (*SD*)	*M* (*SD*)	*Estimate*	*RR*	*0.5% CI*	*99.5% CI*
Use Frequency Last 30 Days	5.65 (7.78)	11.30 (10.51)	0.66	**1.94**	**1.65**	**2.29**
Typical Quantity*	2.86 (5.82)	5.99 (8.81)	0.68	**0.98**	**0.66**	**1.36**
Typical Frequency	3.11 (3.94)	6.67 (7.75)	0.74	**2.10**	**1.76**	**2.49**
***Marijuana-related Consequences***	*M* (*SD*)	*M* (*SD*)	*Estimate*	*RR*	*0.5% CI*	*99.5% CI*
B-MACQ – Total Score	2.01 (3.10)	3.93 (4.31)	0.42	**1.52**	**1.26**	**1.83**

*Note*: *For alcohol and marijuana quantity, values were logged transformed within the regression models and estimates were exponentiated and then 1 was subtracted from the result to create a predicted percent change similar to a Rate Ratio. RR = Rate Ratio, Significant results are bolded and were determined via 99% CIs for the exponentiated estimates that did not contain 0 and Rate Ratios that did not contain 1. Regression models controlled for age and gender (estimates available upon request). For B-YAACQ analyses, typical alcohol frequency was also added as a covariate. For B-MACQ analyses, typical marijuana frequency was also added as a covariate.

In examining effects within countries, patterns were consistent to what was found in the total sample, such that individuals who engaged in SAM use largely reported more alcohol and marijuana use and related negative consequences compared to those who reported CAM use (see Supplemental Tables 1–7). In examining effects across countries, the RRs were quite similar; however, there were differences in the patterns of statistical significance, which were likely due to sample size differences between countries. For example, the RRs in Uruguay were larger than the total analytic sample for a number of indicators, but were not statistically significant due to the small number of participants from Uruguay in the study.

## 4 Discussion

The aim of the present research was to study CAM and SAM use prevalence among young adult college students from seven different countries and examine if CAM vs SAM use status predicted different alcohol and marijuana related outcomes. We also explored if those associations were consistent across countries from three different continents.

Descriptive results showed that among college students that reported lifetime use of both alcohol and marijuana, students from the U.S., Canada, South Africa, Argentina, and Uruguay reported a higher prevalence of consuming both drugs during the last 30 days compared to only consuming alcohol. Comparably, students from Spain and England reported a higher prevalence of alcohol only consumption compared to use of both drugs. These differences could be explained in part by the current marijuana policies in Spain and England (see Table 1), which have higher access restrictions than the other countries sampled. In fact, among all the countries included in the research, the only country in which the recreational use of marijuana is completely illegal is in England. Similarly, in Spain the marijuana consumption is only permitted in the private sphere.

Among students who reported both alcohol and marijuana use during the last 30 days, the results showed similar prevalence rates of SAM vs CAM (∼75% vs 25%, respectively) that have been found in a previous sample of U.S. students (76.9% SAM vs 23.1% CAM use; Looby et al., 2021). Moreover, roughly 75% (or higher) of students reported SAM use vs CAM use across all the countries. These results confirm that in addition to the U.S., SAM use is typical among college students that consume both substances. Moreover, our results showed that prevalence rates of SAM (vs CAM) were higher in South America (i.e., Uruguay and Argentina) than the rest of the countries. Previous reports have shown that prevalence rates of marijuana use are usually higher in countries like the U.S. than in South-America, but they also show that marijuana use has remained relatively stable in U.S. (Miech et al., 2018) whereas South-American countries are showing steady increases (Schleimer et al., 2019). The fact that the data of the current research was collected recently (i.e., 2019–2020), the higher SAM reported in Argentina and Uruguay could be influenced by the increased tendency and higher acceptance of the marijuana consumption in both countries, which could lead to more prejudicial drug patterns, including SAM use. Moreover, although in Uruguay recreational and therapeutic use are permitted, they are not totally regulated, which could also influence in the development of worsen drug use patterns like SAM use. In any case, as the sample size of Uruguay undergraduates was the lowest in the study, results should be taken in caution, and should be replicated in future research.

In addition, and in line with previous studies performed in U.S., the sample composed by students that endorsed SAM use from the seven countries reported higher marijuana and alcohol use and more negative consequences than students endorsing CAM use (Cummings et al., 2019, Jackson et al., 2020, Looby et al., 2021, Sokolovsky et al., 2020). Our results suggest that the alcohol—marijuana consumption patterns and consequences associated with SAM (vs CAM) found in previous studies within U.S. can be expected in young adult college students from other countries. In fact, when the associations between the SAM and CAM status and alcohol/marijuana outcomes were explored in each country separately, a similar tendency among all countries was found. The only few differences were related to statistical significance but this could be explained by the low sample size of some countries (i.e., England and Uruguay).

### 4.1 Limitations

Despite the numerous strengths of the current multi-country study, there are some notable limitations. First, the cross-sectional nature of the study design precludes us from making any causal inferences about the role that co-use status (SAM vs. CAM) plays in risk for alcohol and marijuana related harms. Future studies should use longitudinal designs to establish definitive temporal associations between SAM/CAM use and substance-related harms among young people. Second, while the overall sample size was very large, some country-specific subsamples were small (i.e., Uruguay and England). This may have negatively impacted our statistical power and subsequently, may explain some differences in effects between countries. Third, our study focused on a specific population (i.e., college students). It will be important for generalizability to expand the current models to other populations (e.g., community samples, clinical samples, non-college attending young adults).

### 4.2 Conclusions and future directions

Many theories claim that simultaneous use of substances confers unique risk for dependence formation. For example, individuals with lower socioeconomic status are at greater risk of alcohol dependence despite consuming less alcohol, and this paradox is thought to be at least partially explained by co-use of alcohol with other substances such as tobacco increasing the reward value of alcohol thus promoting dependence formation (Bellis et al., 2016). Similarly, theorists exploring the gateway hypothesis, commonly explain the risk of transitioning from one substance to another as due to the enhanced reward value of the second drug when co-used with the first (Moss et al., 2014). Finally, animal research has confirmed these claims by showing that experimenter administration of one drug enhances the rewarding potential of the other, self-administered drug. This additive effect on that self-administered drug’s reward value arguably confers risk of dependence formation to that drug (Crummy et al., 2020). The converging evidence from these fields suggests that simultaneous substance use could play a more fundamental/general role in dependence formation than previous considered. The present study supports this claim by demonstrating that the risk of negative consequences conferred by simultaneous substance use is generalizable across multiple countries.

The current findings also have implications for screening protocols used to identify individuals at risk of dependence and other substance related harms. Screening protocols in experimental and intervention studies commonly measure single substance use severity as the marker for risk. Where multiple substance use is measured, analytical methods are rarely employed to index co-use as a marker. Moreover, few studies include the necessary items to discriminate simultaneous versus concurrent use. Given the current finding that simultaneous use is a unique risk marker, future studies should incorporate the sort of screening items and analytical methods described here to achieve greater resolution in the quantification of individual risk.

## 5 Role of funding sources

Dr. Bravo was supported by a training grant (T32-AA018108) from the National Institute on Alcohol Abuse and Alcoholism (NIAAA) in the United States during the duration of data collection for this project. Data collection was supported, in part, by grant T32-AA018108. NIAAA had no role in the study design, collection, analysis or interpretation of the data, writing the manuscript, or the decision to submit the paper for publication. Data collection in Spain was also supported by grants UJI-A2019-08 from the Universitat Jaume I and grant PSI2015-67766-R from the Spanish Ministry of Economy and Competitiveness (MINECO). Data collection in Argentina was also supported by grants from the National Secretary of Science and Technology (FONCYT, grant number PICT 2018-03170) and by grants from the Secretary of Science and Technology- National University of Córdoba (SECyT-UNC).

## 6 Disclosures

The authors report no conflict of interest.

## 7 Data Availability Statement

Data and analytic outputs are available at DOI https://doi.org/10.17605/OSF.IO/KATU9.

## CRediT authorship contribution statement

**Adrian J. Bravo:** Conceptualization, Funding acquisition, Investigation, Methodology, Project administration, Resources, Supervision, Software, Writing – original draft. **Mark A. Prince:** Methodology, Software, Investigation, Resources, Writing – original draft. **Angelina Pilatti:** Investigation, Resources, Writing – original draft, Funding acquisition. **Laura Mezquita:** Investigation, Resources, Writing – original draft, Funding acquisition. **Matthew T. Keough:** Investigation, Resources, Writing – original draft. **Lee Hogarth:** Investigation, Resources, Writing – original draft. **:** .

## Declaration of Competing Interest

The authors declare that they have no known competing financial interests or personal relationships that could have appeared to influence the work reported in this paper.
