# Supplementary material for: Young adult concurrent use and simultaneous use of alcohol and marijuana: A cross-national examination among college students in seven countries
Source: Addict Behav Rep. 2021 Sep 4;14:100373. doi: 10.1016/j.abrep.2021.100373 (PMC8664774; doi:10.1016/j.abrep.2021.100373)
Supplement: Supplementary data 1 [file mmc1.docx]

Supplemental Table 1

*Negative Binomial regression models among those reporting past 30-day alcohol & marijuana concurrent use vs. simultaneous use in USA sample.*

|  | Concurrent Use  (*n* = 294) | Simultaneous Use  (*n* = 835) | Negative Binomial Regression Models Results  (0 = concurrent; 1 = simultaneous) | | | |
| --- | --- | --- | --- | --- | --- | --- |
| ***Alcohol Use Indicators*** | *M* (*SD*) | *M* (*SD*) | *Estimate* | *RR* | *0.5% CI* | *99.5% CI* |
| Use Frequency Last 30 Days | 5.12 (4.79) | 6.78 (5.37) | 0.26 | **1.3** | **1.11** | **1.53** |
| Drunk Frequency Last 30 Days | 2.95 (3.49) | 3.99 (3.63) | 0.3 | **1.34** | **1.11** | **1.63** |
| Sick from Drinking Frequency Last 30 Days | 0.43 (1.96) | 0.58 (1.15) | 0.29 | 1.33 | 0.68 | 2.62 |
| Binge Frequency Last 30 Days | 2.41 (3.43) | 3.29 (3.62) | 0.27 | **1.31** | **1.04** | **1.66** |
| Typical Quantity* | 120.94 (106.06) | 177.31 (141.21) | 0.4 | **0.50** | **0.27** | **0.77** |
| Typical Frequency | 3.00 (2.34) | 4.24 (3.42) | 0.32 | **1.38** | **1.19** | **1.60** |
| ***Alcohol-related Consequences*** | *M* (*SD*) | *M* (*SD*) | *Estimate* | *RR* | *0.5% CI* | *99.5% CI* |
| B-YAACQ – Total Score | 4.40 (4.06) | 6.17 (4.76) | 0.69 | **2.00** | **1.73** | **2.30** |
| ***Marijuana Use Indicators*** | *M* (*SD*) | *M* (*SD*) | *Estimate* | *OR* | *0.5% CI* | *99.5% CI* |
| Use Frequency Last 30 Days | 6.05 (8.12) | 13.08 (10.96) | 0.74 | **2.10** | **1.7** | **2.61** |
| Typical Quantity* | 2.90 (5.60) | 7.46 (10.13) | 0.84 | **1.31** | **0.82** | **1.94** |
| Typical Frequency | 3.47 (4.46) | 8.11 (8.95) | 0.83 | **2.29** | **1.82** | **2.87** |
| ***Marijuana-related Consequences*** | *M* (*SD*) | *M* (*SD*) | *Estimate* | *RR* | *0.5% CI* | *99.5% CI* |
| B-MACQ – Total Score | 2.11 (3.29) | 4.12 (4.52) | 0.43 | **1.53** | **1.18** | **1.99** |

*Note*: *For alcohol and marijuana quantity, values were logged transformed within the regression models and estimates were exponentiated and then 1 was subtracted from the result to create a predicted percent change similar to a Rate Ratio. RR = Rate Ratio, Significant results are bolded and were determined via 99% CIs for the exponentiated estimates that did not contain 0 and Rate Ratios that did not contain 1. Regression models controlled for age and gender (estimates available upon request). For B-YAACQ analyses, typical alcohol frequency was also added as a covariate. For B-MACQ analyses, typical marijuana frequency was also added as a covariate.

Supplemental Table 2

*Negative Binomial regression models among those reporting past 30-day alcohol & marijuana concurrent use vs. simultaneous use in Canada sample.*

|  | Concurrent Use  (*n* = 89) | Simultaneous Use  (*n* = 246) | Negative Binomial Regression Models Results  (0 = concurrent; 1 = simultaneous) | | | |
| --- | --- | --- | --- | --- | --- | --- |
| ***Alcohol Use Indicators*** | *M* (*SD*) | *M* (*SD*) | *Estimate* | *RR* | *0.5% CI* | *99.5% CI* |
| Use Frequency Last 30 Days | 4.64 (4.36) | 5.86 (4.72) | 0.25 | 1.28 | 0.96 | 1.71 |
| Drunk Frequency Last 30 Days | 2.67 (3.44) | 3.14 (2.98) | 0.20 | 1.22 | 0.85 | 1.75 |
| Sick from Drinking Frequency Last 30 Days | 0.69 (2.78) | 0.48 (0.97) | -0.18 | 0.84 | 0.3 | 2.34 |
| Binge Frequency Last 30 Days | 2.63 (3.96) | 2.85 (3.23) | 0.11 | 1.11 | 0.73 | 1.70 |
| Typical Quantity* | 145.46 (115.81) | 179.60 (137.34) | 0.22 | 0.24 | -0.05 | 0.63 |
| Typical Frequency | 3.13 (2.82) | 3.83 (2.61) | 0.19 | 1.21 | 0.92 | 1.60 |
| ***Alcohol-related Consequences*** | *M* (*SD*) | *M* (*SD*) | *Estimate* | *RR* | *0.5% CI* | *99.5% CI* |
| B-YAACQ – Total Score | 4.82 (3.98) | 6.05 (4.56) | 0.79 | **2.20** | **1.80** | **2.69** |
| ***Marijuana Use Indicators*** | *M* (*SD*) | *M* (*SD*) | *Estimate* | *RR* | *0.5% CI* | *99.5% CI* |
| Use Frequency Last 30 Days | 5.57 (7.59) | 9.13 (9.86) | 0.43 | **1.54** | **1.02** | **2.33** |
| Typical Quantity* | 2.07 (2.48) | 3.82 (5.59) | 0.47 | **0.60** | **0.08** | **1.37** |
| Typical Frequency | 2.65 (2.73) | 4.63 (5.62) | 0.54 | **1.71** | **1.16** | **2.52** |
| ***Marijuana-related Consequences*** | *M* (*SD*) | *M* (*SD*) | *Estimate* | *RR* | *0.5% CI* | *99.5% CI* |
| B-MACQ – Total Score | 2.02 (3.18) | 3.53 (4.16) | 0.35 | 1.42 | 0.88 | 2.31 |

*Note*: *For alcohol and marijuana quantity, values were logged transformed within the regression models and estimates were exponentiated and then 1 was subtracted from the result to create a predicted percent change similar to a Rate Ratio. RR = Rate Ratio, Significant results are bolded and were determined via 99% CIs for the exponentiated estimates that did not contain 0 and Rate Ratios that did not contain 1. Regression models controlled for age and gender (estimates available upon request). For B-YAACQ analyses, typical alcohol frequency was also added as a covariate. For B-MACQ analyses, typical marijuana frequency was also added as a covariate.

.

Supplemental Table 3

*Negative Binomial regression models among those reporting past 30-day alcohol & marijuana concurrent use vs. simultaneous use in South Africa sample.*

|  | Concurrent Use  (*n* = 45) | Simultaneous Use  (*n* = 148) | Negative Binomial Regression Models Results  (0 = concurrent; 1 = simultaneous) | | | |
| --- | --- | --- | --- | --- | --- | --- |
| ***Alcohol Use Indicators*** | *M* (*SD*) | *M* (*SD*) | *Estimate* | *RR* | *0.5% CI* | *99.5% CI* |
| Use Frequency Last 30 Days | 5.67 (4.92) | 8.71 (5.68) | 0.42 | **1.52** | **1.06** | **2.19** |
| Drunk Frequency Last 30 Days | 1.82 (1.83) | 4.70 (4.58) | 0.94 | **2.57** | **1.65** | **3.99** |
| Sick from Drinking Frequency Last 30 Days | 0.39 (1.06) | 0.99 (2.35) | 0.98 | 2.66 | 0.85 | 8.35 |
| Binge Frequency Last 30 Days | 1.89 (1.81) | 4.47 (5.23) | 0.87 | **2.38** | **1.53** | **3.71** |
| Typical Quantity* | 91.05 (58.04) | 130.64 (111.69) | 0.29 | 0.34 | -0.03 | 0.85 |
| Typical Frequency | 3.86 (2.43) | 5.27 (4.07) | 0.32 | **1.37** | **1.01** | **1.87** |
| ***Alcohol-related Consequences*** | *M* (*SD*) | *M* (*SD*) | *Estimate* | *RR* | *0.5% CI* | *99.5% CI* |
| B-YAACQ – Total Score | 5.77 (4.15) | 7.90 (4.58) | 0.63 | **1.88** | **1.47** | **2.40** |
| ***Marijuana Use Indicators*** | *M* (*SD*) | *M* (*SD*) | *Estimate* | *RR* | *0.5% CI* | *99.5% CI* |
| Use Frequency Last 30 Days | 4.84 (7.32) | 12.13 (10.46) | 0.92 | **2.5** | **1.37** | **4.56** |
| Typical Quantity* | 4.20 (9.50) | 7.41 (9.26) | 0.79 | **1.21** | **0.26** | **2.87** |
| Typical Frequency | 3.05 (4.50) | 7.42 (7.57) | 0.92 | **2.5** | **1.40** | **4.46** |
| ***Marijuana-related Consequences*** | *M* (*SD*) | *M* (*SD*) | *Estimate* | *RR* | *0.5% CI* | *99.5% CI* |
| B-MACQ – Total Score | 1.91 (2.79) | 5.10 (4.11) | 0.66 | **1.93** | **1.15** | **3.23** |

*Note*: *For alcohol and marijuana quantity, values were logged transformed within the regression models and estimates were exponentiated and then 1 was subtracted from the result to create a predicted percent change similar to a Rate Ratio. RR = Rate Ratio, Significant results are bolded and were determined via 99% CIs for the exponentiated estimates that did not contain 0 and Rate Ratios that did not contain 1. Regression models controlled for age and gender (estimates available upon request). For B-YAACQ analyses, typical alcohol frequency was also added as a covariate. For B-MACQ analyses, typical marijuana frequency was also added as a covariate.

Supplemental Table 4

*Negative Binomial regression models among those reporting past 30-day alcohol & marijuana concurrent use vs. simultaneous use in Spain sample.*

|  | Concurrent Use  (*n* = 24) | Simultaneous Use  (*n* = 77) | Negative Binomial Regression Models Results  (0 = concurrent; 1 = simultaneous) | | | |
| --- | --- | --- | --- | --- | --- | --- |
| ***Alcohol Use Indicators*** | *M* (*SD*) | *M* (*SD*) | *Estimate* | *RR* | *0.5% CI* | *99.5% CI* |
| Use Frequency Last 30 Days | 4.04 (2.22) | 7.08 (5.73) | 0.5 | **1.65** | **1.12** | **2.43** |
| Drunk Frequency Last 30 Days | 1.79 (2.19) | 3.16 (4.35) | 0.7 | 2.01 | 0.91 | 4.44 |
| Sick from Drinking Frequency Last 30 Days | 0.92 (1.98) | 0.62 (1.20) | -0.06 | 0.94 | 0.27 | 3.31 |
| Binge Frequency Last 30 Days | 1.46 (1.96) | 2.35 (2.83) | 0.54 | 1.71 | 0.78 | 3.74 |
| Typical Quantity* | 105.23 (116.84) | 152.06 (116.98) | 0.65 | **0.91** | **0.05** | **2.47** |
| Typical Frequency | 2.50 (1.95) | 4.40 (2.79) | 0.53 | **1.7** | **1.08** | **2.68** |
| ***Alcohol-related Consequences*** | *M* (*SD*) | *M* (*SD*) | *Estimate* | *RR* | *0.5% CI* | *99.5% CI* |
| B-YAACQ – Total Score | 5.25 (5.46) | 6.09 (4.32) | 0.77 | **2.17** | **1.64** | **2.87** |
| ***Marijuana Use Indicators*** | *M* (*SD*) | *M* (*SD*) | *Estimate* | *RR* | *0.5% CI* | *99.5% CI* |
| Use Frequency Last 30 Days | 6.46 (8.35) | 8.68 (9.86) | 0.14 | 1.15 | 0.56 | 2.37 |
| Typical Quantity* | 1.31 (1.84) | 3.70 (5.94) | 0.78 | **1.18** | **0.09** | **3.37** |
| Typical Frequency | 3.70 (5.94) | 2.50 (2.42) | 0.36 | 1.44 | 0.85 | 2.43 |
| ***Marijuana-related Consequences*** | *M* (*SD*) | *M* (*SD*) | *Estimate* | *RR* | *0.5% CI* | *99.5% CI* |
| B-MACQ – Total Score | 1.92 (2.22) | 4.31 (4.46) | 0.36 | 1.44 | 0.77 | 2.69 |

*Note*: *For alcohol and marijuana quantity, values were logged transformed within the regression models and estimates were exponentiated and then 1 was subtracted from the result to create a predicted percent change similar to a Rate Ratio. RR = Rate Ratio, Significant results are bolded and were determined via 99% CIs for the exponentiated estimates that did not contain 0 and Rate Ratios that did not contain 1. Regression models controlled for age and gender (estimates available upon request). For B-YAACQ analyses, typical alcohol frequency was also added as a covariate. For B-MACQ analyses, typical marijuana frequency was also added as a covariate.

Supplemental Table 5

*Negative Binomial regression models among those reporting past 30-day alcohol & marijuana concurrent use vs. simultaneous use in Argentina sample.*

|  | Concurrent Use  (*n* = 45) | Simultaneous Use  (*n* = 221) | Negative Binomial Regression Models Results  (0 = concurrent; 1 = simultaneous) | | | |
| --- | --- | --- | --- | --- | --- | --- |
| ***Alcohol Use Indicators*** | *M* (*SD*) | *M* (*SD*) | *Estimate* | *RR* | *0.5% CI* | *99.5% CI* |
| Use Frequency Last 30 Days | 5.49 (4.98) | 7.40 (4.88) | 0.28 | 1.33 | 0.92 | 1.9 |
| Drunk Frequency Last 30 Days | 0.82 (1.15) | 1.99 (2.39) | 0.87 | **2.38** | **1.34** | **4.22** |
| Sick from Drinking Frequency Last 30 Days | 0.27 (0.72) | 0.63 (1.34) | 0.87 | 2.38 | 0.8 | 7.06 |
| Binge Frequency Last 30 Days | 0.93 (1.29) | 2.17 (2.86) | 0.76 | **2.14** | **1.22** | **3.75** |
| Typical Quantity* | 123.64 (121.72) | 149.80 (124.60) | 0.25 | 0.28 | -0.11 | 0.84 |
| Typical Frequency | 3.86 (4.23) | 4.33 (3.09) | 0.1 | 1.11 | 0.71 | 1.71 |
| ***Alcohol-related Consequences*** | *M* (*SD*) | *M* (*SD*) | *Estimate* | *RR* | *0.5% CI* | *99.5% CI* |
| B-YAACQ – Total Score | 3.45 (2.55) | 5.61 (4.16) | 0.8 | **2.22** | **1.82** | **2.70** |
| ***Marijuana Use Indicators*** | *M* (*SD*) | *M* (*SD*) | *Estimate* | *RR* | *0.5% CI* | *99.5% CI* |
| Use Frequency Last 30 Days | 3.84 (5.98) | 8.81 (8.72) | 0.8 | **2.24** | **1.19** | **4.19** |
| Typical Quantity* | 3.99 (8.75) | 3.11 (5.17) | 0.4 | 0.49 | -0.25 | 1.98 |
| Typical Frequency | 2.32 (2.63) | 4.62 (5.09) | 0.69 | **2.00** | **1.24** | **3.21** |
| ***Marijuana-related Consequences*** | *M* (*SD*) | *M* (*SD*) | *Estimate* | *RR* | *0.5% CI* | *99.5% CI* |
| B-MACQ – Total Score | 1.73 (2.79) | 3.22 (3.78) | 0.31 | 1.36 | 0.77 | 2.41 |

*Note*: *For alcohol and marijuana quantity, values were logged transformed within the regression models and estimates were exponentiated and then 1 was subtracted from the result to create a predicted percent change similar to a Rate Ratio. RR = Rate Ratio, Significant results are bolded and were determined via 99% CIs for the exponentiated estimates that did not contain 0 and Rate Ratios that did not contain 1. Regression models controlled for age and gender (estimates available upon request). For B-YAACQ analyses, typical alcohol frequency was also added as a covariate. For B-MACQ analyses, typical marijuana frequency was also added as a covariate.

Supplemental Table 6

*Negative Binomial regression models among those reporting past 30-day alcohol & marijuana concurrent use vs. simultaneous use in Uruguay sample.*

|  | Concurrent Use  (*n* = 5) | Simultaneous Use  (*n* = 37) | Negative Binomial Regression Models Results  (0 = concurrent; 1 = simultaneous) | | | |
| --- | --- | --- | --- | --- | --- | --- |
| ***Alcohol Use Indicators*** | *M* (*SD*) | *M* (*SD*) | *Estimate* | *RR* | *0.5% CI* | *99.5% CI* |
| Use Frequency Last 30 Days | 4.80 (3.11) | 6.30 (4.82) | 0.36 | 1.43 | 0.72 | 2.84 |
| Drunk Frequency Last 30 Days | 0.60 (1.34) | 1.24 (2.03) | 0.75 | 2.11 | 0.21 | 20.76 |
| Sick from Drinking Frequency Last 30 Days | 0.20 (0.45) | 0.65 (1.83) | 1.45 | 4.26 | 0.46 | 39.85 |
| Binge Frequency Last 30 Days | 1.20 (1.64) | 1.24 (1.48) | 0.04 | 1.05 | 0.27 | 4.00 |
| Typical Quantity* | 54.00 (54.59) | 104.44 (88.82) | 0.65 | 0.92 | -0.25 | 3.90 |
| Typical Frequency | 1.60 (0.55) | 3.56 (2.06) | 0.8 | **2.22** | **1.47** | **3.35** |
| ***Alcohol-related Consequences*** | *M* (*SD*) | *M* (*SD*) | *Estimate* | *RR* | *0.5% CI* | *99.5% CI* |
| B-YAACQ – Total Score | 3.80 (3.90) | 3.27 (3.36) | 0.67 | 1.95 | 0.96 | 3.98 |
| ***Marijuana Use Indicators*** | *M* (*SD*) | *M* (*SD*) | *Estimate* | *RR* | *0.5% CI* | *99.5% CI* |
| Use Frequency Last 30 Days | 8.60 (12.28) | 11.27 (10.39) | 0.5 | 1.65 | 0.43 | 6.29 |
| Typical Quantity* | 2.03 (1.50) | 7.54 (10.41) | 0.77 | 1.16 | -0.20 | 4.82 |
| Typical Frequency | 2.00 (0.71) | 6.58 (5.75) | 1.11 | **3.03** | **1.57** | **5.85** |
| ***Marijuana-related Consequences*** | *M* (*SD*) | *M* (*SD*) | *Estimate* | *RR* | *0.5% CI* | *99.5% CI* |
| B-MACQ – Total Score | 1.20 (0.45) | 3.89 (4.61) | 0.31 | 1.36 | 0.53 | 3.48 |

*Note*: *For alcohol and marijuana quantity, values were logged transformed within the regression models and estimates were exponentiated and then 1 was subtracted from the result to create a predicted percent change similar to a Rate Ratio. RR = Rate Ratio, Significant results are bolded and were determined via 99% CIs for the exponentiated estimates that did not contain 0 and Rate Ratios that did not contain 1. Regression models controlled for age and gender (estimates available upon request). For B-YAACQ analyses, typical alcohol frequency was also added as a covariate. For B-MACQ analyses, typical marijuana frequency was also added as a covariate.

Supplemental Table 7

*Negative Binomial regression models among those reporting past 30-day alcohol & marijuana concurrent use vs. simultaneous use in England sample.*

|  | Concurrent Use  (*n* = 12) | Simultaneous Use  (*n* = 46) | Negative Binomial Regression Models Results  (0 = concurrent; 1 = simultaneous) | | | |
| --- | --- | --- | --- | --- | --- | --- |
| ***Alcohol Use Indicators*** | *M* (*SD*) | *M* (*SD*) | *Estimate* | *RR* | *0.5% CI* | *99.5% CI* |
| Use Frequency Last 30 Days | 9.17 (6.22) | 11.50 (5.93) | 0.18 | 1.2 | 0.75 | 1.91 |
| Drunk Frequency Last 30 Days | 5.67 (4.81) | 6.26 (4.43) | 0.15 | 1.16 | 0.68 | 1.99 |
| Sick from Drinking Frequency Last 30 Days | 1.08 (1.73) | 0.94 (1.78) | -0.22 | 0.81 | 0.23 | 2.83 |
| Binge Frequency Last 30 Days | 3.33 (3.94) | 5.94 (4.75) | 0.56 | 1.74 | 0.78 | 3.90 |
| Typical Quantity* | 119.33 (80.18) | 188.67 (128.83) | 0.39 | 0.48 | -0.19 | 1.73 |
| Typical Frequency | 4.83 (3.04) | 6.63 (3.73) | 0.28 | 1.32 | 0.83 | 2.11 |
| ***Alcohol-related Consequences*** | *M* (*SD*) | *M* (*SD*) | *Estimate* | *RR* | *0.5% CI* | *99.5% CI* |
| B-YAACQ – Total Score | 7.50 (5.99) | 9.57 (4.64) | -0.55 | **0.58** | **0.45** | **0.75** |
| ***Marijuana Use Indicators*** | *M* (*SD*) | *M* (*SD*) | *Estimate* | *RR* | *0.5% CI* | *99.5% CI* |
| Use Frequency Last 30 Days | 3.42 (3.32) | 4.28 (6.01) | 0.25 | 1.29 | 0.47 | 3.50 |
| Typical Quantity* | 2.21 (2.30) | 3.43 (5.41) | 0.18 | 0.2 | -0.39 | 1.33 |
| Typical Frequency | 2.46 (2.07) | 3.27 (4.91) | 0.53 | 1.71 | 0.7 | 4.13 |
| ***Marijuana-related Consequences*** | *M* (*SD*) | *M* (*SD*) | *Estimate* | *RR* | *0.5% CI* | *99.5% CI* |
| B-MACQ – Total Score | 1.58 (2.23) | 1.85 (2.30) | 0.16 | 1.17 | 0.49 | 2.81 |

*Note*: *For alcohol and marijuana quantity, values were logged transformed within the regression models and estimates were exponentiated and then 1 was subtracted from the result to create a predicted percent change similar to a Rate Ratio. RR = Rate Ratio, Significant results are bolded and were determined via 99% CIs for the exponentiated estimates that did not contain 0 and Rate Ratios that did not contain 1. Regression models controlled for age and gender (estimates available upon request). For B-YAACQ analyses, typical alcohol frequency was also added as a covariate. For B-MACQ analyses, typical marijuana frequency was also added as a covariate.
